# Quality and guideline adherence of child-oriented toothbrushing videos on YouTube: a comparative study of Turkish and English content

**DOI:** 10.1186/s12903-026-08607-w

**Published:** 2026-05-21

**Authors:** Merter Güçlü, Hatice Selin Güçlü

**Affiliations:** 1 Department of Periodontology, Faculty of Dentistry, Yozgat Bozok University, Yozgat, Turkey; 2 Vocational School of Health Services, Oral and Dental Health Program, Ankara Medipol University, Ankara, Turkey

**Keywords:** Pediatric dentistry, Health information quality, Toothbrushing, Social media, YouTube

## Abstract

**Background:**

Early childhood caries remains a major global public health concern. As parents and children increasingly rely on digital platforms for health information, the quality and reliability of online educational resources become critical. Despite the abundance of general oral health content, studies specifically evaluating child-oriented toothbrushing instructions remain scarce. To address this gap, the present study evaluated and compared the quality, reliability, and guideline adherence of child-oriented toothbrushing videos in Turkish and English, highlighting potential disparities between global and localized digital health information ecosystems.

**Methods:**

A cross-sectional analysis of YouTube videos was conducted using predefined Turkish and English keywords. After applying exclusion criteria (e.g., entertainment-only and non-instructional videos), 51 videos (27 Turkish, 24 English) were included. Two independent reviewers assessed videos using the Global Quality Scale (GQS), DISCERN, and a 10-item guideline-based content compliance checklist. Engagement metrics (views, likes, viewing rate, and interaction index) were recorded, and associations between engagement and evaluation scores were examined.

**Results:**

English-language videos showed higher median GQS and DISCERN scores than Turkish-language videos (3.25 vs. 1.50, *p* = 0.042; and 3.00 vs. 2.00, *p* = 0.037, respectively). Guideline-based compliance scores were also higher in English videos (5.50 vs. 3.00, *p* = 0.018). Videos uploaded by animation channels demonstrated significantly lower quality and compliance scores compared with educational and health-related channels (*p* < 0.001). No significant correlations were observed between engagement metrics and GQS, DISCERN, or compliance scores (all *p* > 0.05).

**Conclusion:**

The overall quality, reliability, and guideline adherence of child-oriented toothbrushing videos were limited, and Turkish-language content demonstrated lower evaluation scores than English-language content. The lack of association between engagement and evaluation scores indicates that popularity does not reflect informational quality, underscoring the need for greater involvement of dental professionals in producing evidence-based, age-appropriate digital educational materials. Actionable strategies, such as clinician-guided video recommendations and the future development of digital quality-labeling systems, may help guide families toward more reliable content.

## Background

Early childhood caries (ECC), defined as the presence of at least one decayed, missing, or filled primary tooth in children under five years of age, is among the most common oral health problems in early childhood [1]. ECC may cause pain, infection, functional impairment, and psychosocial consequences [2, 3], and its prevalence is particularly high in socioeconomically disadvantaged populations [4]. Globally, dental caries remains one of the most prevalent chronic diseases affecting children’s quality of life [5], and the World Health Organization’s 2022 Global Oral Health Status Report identifies ECC as a continuing major public health concern [6]. Although ECC is defined for children under five years of age, early caries experience is widely recognized as a strong predictor of increased caries risk in later childhood [2].

Oral hygiene behaviors established during childhood play a crucial role in long-term oral health outcomes. Inadequate or improper toothbrushing contributes to dental plaque accumulation and insufficient fluoride exposure, both of which are central etiological factors in the development and progression of ECC [3, 7]. Professional guidelines recommend initiating oral hygiene practices with the eruption of the first tooth, using fluoridated toothpaste, and maintaining parental supervision during early years to ensure effective plaque control and appropriate fluoride use [8]. Therefore, the scientific accuracy and guideline consistency of toothbrushing education directed at children are critically important for early caries prevention. The early school years represent a transitional period from parental supervision toward increasing self-care responsibility, and the establishment of proper toothbrushing behaviors during this stage plays a pivotal role in reducing long-term caries susceptibility [9].

Digital media increasingly influences children’s learning processes and health-related behaviors, including oral hygiene practices. While high-quality content may support positive behavioral development, low-quality or inaccurate information may promote inadequate oral hygiene practices [10–12]. YouTube is one of the most widely used digital platforms worldwide and lacks professional editorial oversight [13]. Previous studies have reported that pediatric oral health content on YouTube often demonstrates limited educational quality and insufficient adherence to professional guidelines [13–15]. However, studies specifically evaluating toothbrushing instructional content directed at children remain limited, and Turkish-language content has similarly been reported to exhibit low quality and poor guideline compliance [16, 17].

Accordingly, this study aimed to evaluate the scientific accuracy, educational quality, reliability, and guideline adherence of toothbrushing videos intended for children on YouTube and to compare these characteristics between Turkish- and English-language content. English was selected as it serves as the global lingua franca, representing a vast, internationally targeted content ecosystem, whereas Turkish represents a localized non-English digital health information ecosystem. This comparison is relevant for highlighting potential disparities in global access to high-quality oral health information based on linguistic and regional production capacities. Although ECC is more prevalent in younger children, independent toothbrushing behaviors are typically established during the early school years; therefore, this study focused on children aged 6–12 years. It was hypothesized that a substantial proportion of child-oriented toothbrushing videos would demonstrate incomplete adherence to evidence-based guidelines and moderate-to-low educational quality and reliability, and that significant differences would exist between Turkish- and English-language content, as well as among different uploader types.

## Methods

This study was conducted as a cross-sectional and descriptive observational analysis aimed at evaluating the content quality, educational value, and informational reliability of toothbrushing videos designed for children on the YouTube platform. Only publicly accessible videos were included, and no personal data, patient information, or ethically sensitive material was used. Because all examined materials were freely available in the public domain, ethical approval was not required for this study.

### Keyword selection and google trends analysis

Preliminary keyword analysis was performed using Google Trends to identify appropriate Turkish and English search terms. The analysis was conducted using the “Worldwide” region filter and the “Past 5 years” time range to reflect broader global search behavior. Initially, four Turkish terms (“çocuk diş fırçalama,” “çocuklara diş fırçalama eğitimi,” “çocuk diş fırçalama videosu,” and “çocuklar için diş fırçalama”) and four English terms (“kids tooth brushing,” “children brushing teeth,” “tooth brushing for kids,” and “toothbrushing for children”) were compared. Based on the relative stability and consistency of search interest over the selected time period, three Turkish terms (“çocuk diş fırçalama,” “çocuklara diş fırçalama eğitimi,” and “çocuk diş fırçalama videosu”) and three English terms (“kids tooth brushing,” “children brushing teeth,” and “tooth brushing for kids”) were selected as the final study keywords. The terms “çocuklar için diş fırçalama” and “toothbrushing for children” were excluded due to comparatively lower and/or less stable search trends. This approach was adopted to minimize subjectivity in keyword selection and to ensure that the search strategy reflected real-world user search behavior.

### Search strategy and video selection

YouTube searches were conducted in Google Chrome’s incognito mode without logging into any account. To minimize the effect of personalized recommendations, the platform’s default relevance sorting was preserved. No additional filters (e.g., upload date, duration, or channel type) were applied. For each keyword, the first 60 videos sorted by relevance were screened. This approach was adopted because evidence suggests that individuals seeking health information on YouTube rarely navigate beyond the first few pages of search results. Thus, limiting the screening to the first 60 videos closely mimics real-world digital navigation patterns and ensures the evaluation of the most visible and potentially most impactful content, consistent with prior health-related YouTube studies [18]. Duplicate content was identified through URL-based verification, and if a video appeared under multiple search terms, only the first encountered instance was retained. All searches and data recordings (views and likes) were performed on January 20, 2026, and the dataset was fixed on the same day. All retrieved videos were screened according to the predefined inclusion and exclusion criteria, and duplicates were removed before the final eligibility assessment.

### Eligibility criteria

Videos were eligible if they targeted school-aged children (6–12 years), as indicated by the title, description, visual presentation, or language style, and demonstrated active child-performed toothbrushing through direct demonstration, animation-based modeling, or structured verbal instruction. Additional inclusion criteria required a minimum duration of one minute, publication in Turkish or English, and public accessibility on YouTube. Short-form videos (e.g., YouTube Shorts) and videos shorter than one minute were excluded, as their limited duration was considered insufficient for delivering structured, step-by-step instructional content. Videos were also excluded if they depicted caregiver-performed brushing with passive child involvement; were primarily adult-oriented; consisted solely of non-educational music or entertainment content; presented clinical procedures or professional training material; contained explicit product promotion or commercial intent; lacked instructional or demonstrative content related to toothbrushing technique; or represented duplicate or re-uploaded material.

### Data collection and video characteristics

All videos were independently evaluated by two researchers (a periodontist and a pediatric dentist). For each video, the following variables were recorded: title and URL, language (Turkish or English), uploader type (animation channel, educational channel, or health-related channel), video duration, publication date, total number of views, likes, and the number of days between publication and evaluation date. Two normalized engagement metrics were calculated: viewing rate [(total views × 100) / days since publication] and interaction index [(likes × 100) / total views]. Since YouTube no longer publicly displays dislike counts, interaction levels were calculated using available like data only. Both metrics were analyzed as continuous variables to allow comparison across videos with differing publication durations and exposure levels.

### Video content evaluation and reliability assessment

Video content was evaluated using a 10-item guideline-based checklist developed explicitly for this study. The items were systematically derived and adapted from the core recommendations of the American Academy of Pediatric Dentistry (AAPD), the European Academy of Paediatric Dentistry (EAPD), and the World Health Organization (WHO) guidelines on pediatric oral health. Prior to full-scale evaluation, the face and content validity of the checklist were reviewed and supported through a consensus assessment by two independent senior experts (a pediatric dentist and a periodontist) who were not involved in the primary scoring process. The finalized checklist assessed 10 essential components of evidence-based toothbrushing education: brushing frequency, brushing duration, use of fluoridated toothpaste, age-appropriate toothpaste amount, brushing technique, cleaning of all tooth surfaces, tongue/oral cavity cleaning, recommended brushing times, parental supervision, and regular dental visits. Each item was scored dichotomously (1 = present, 0 = absent), a format that may reduce subjective interpretative variability between evaluators compared with Likert-type scoring and may facilitate high inter-rater agreement. Video quality and information reliability were assessed using the Global Quality Scale (GQS) and the DISCERN Short Form [18]. All videos were independently evaluated by two researchers. Prior to formal assessment, a calibration session was conducted to standardize scoring criteria. Inter-rater reliability was analyzed using the Intraclass Correlation Coefficient (ICC) based on a two-way random-effects model with absolute agreement. For checklist-based evaluations, discrepancies were resolved through consensus discussion, whereas the mean of the two independent ratings was used for GQS and DISCERN scores in statistical analyses.

### Statistical analysis

Statistical analyses were performed using IBM SPSS Statistics version 23 (IBM Corp., Armonk, NY, USA). The normality of data distribution was assessed using the Kolmogorov–Smirnov and Shapiro–Wilk tests. For comparisons between two independent groups, the Mann–Whitney U test was applied for variables that did not follow a normal distribution. For comparisons among three or more independent groups, the Kruskal–Wallis test was used, followed by Bonferroni-adjusted Dunn post hoc tests when significant differences were detected. Effect sizes were calculated using rank-biserial correlation (r) for Mann–Whitney U tests and eta-squared (η²) for Kruskal–Wallis tests. Interobserver agreement was evaluated using the Intraclass Correlation Coefficient (ICC). The relationship between non-normally distributed quantitative variables was analyzed using Spearman’s rho correlation coefficient. Quantitative variables were presented as mean ± standard deviation or median (minimum–maximum), as appropriate. Categorical variables were expressed as frequency (percentage). A p-value < 0.05 was considered statistically significant.

## Results

**Fig. 1:**
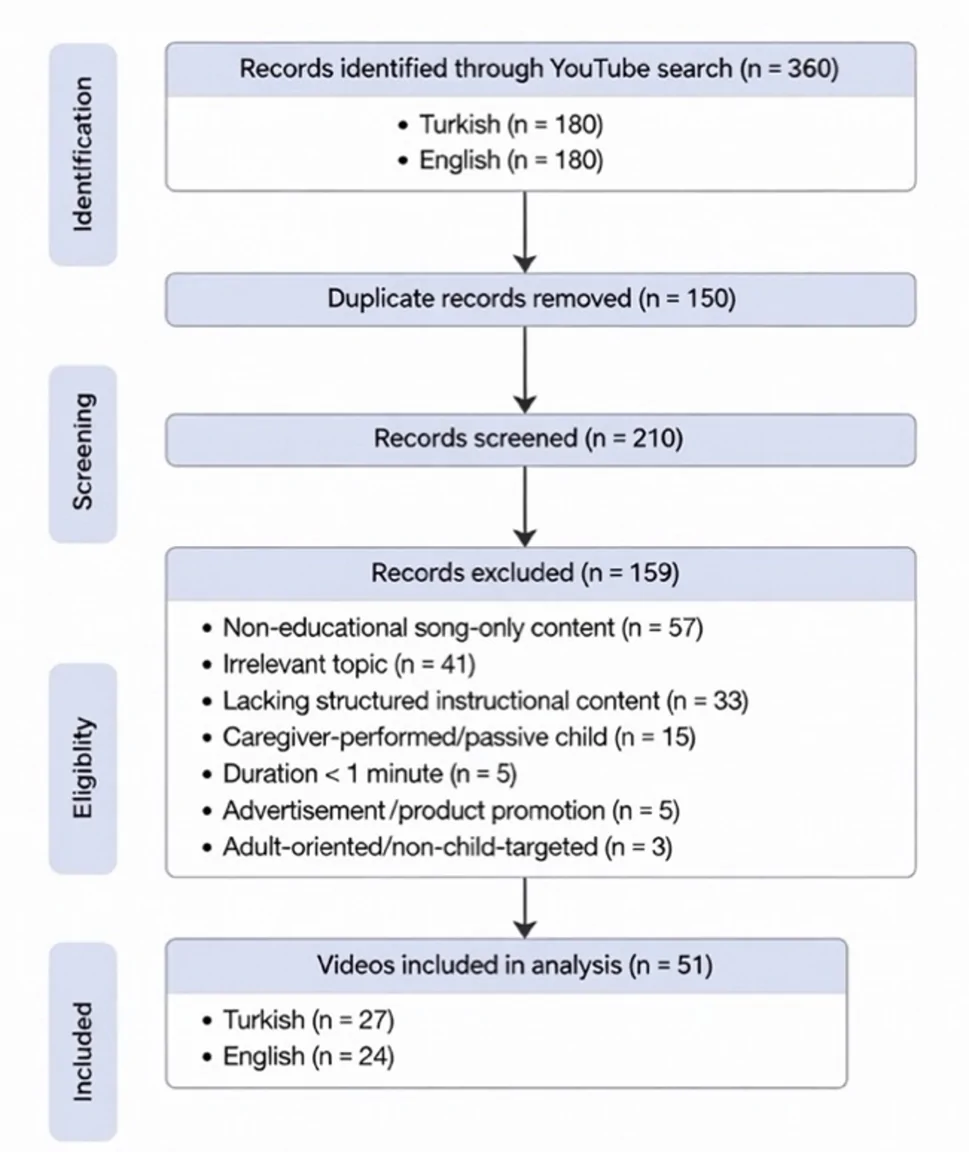
Flow diagram illustrating the video selection process

A total of 360 videos (180 Turkish and 180 English) were initially screened. After removal of duplicate entries through URL-based verification, 210 unique videos remained for eligibility assessment. Following application of the predefined inclusion and exclusion criteria, 51 videos were included in the final analysis (24.3% of the 210 unique videos assessed for eligibility). Of these, 27 were Turkish-language and 24 were English-language videos, while the remaining 159 were excluded. The most frequent reasons for exclusion were non-educational song-only videos without instructional content (*n* = 57), irrelevant topic (*n* = 41), videos mentioning toothbrushing but lacking structured, technique-based instructional content (*n* = 33), caregiver-performed brushing demonstrations with passive child involvement (*n* = 15), videos shorter than one minute (*n* = 5), advertisement or product promotion content (*n* = 5), and adult-oriented or non-child-targeted materials (*n* = 3). The video selection process is illustrated in Fig. 1.

The general characteristics and engagement metrics of the included videos are presented in Table 1. Most videos originated from Türkiye (52.9%), followed by the United States (31.4%), the United Kingdom (7.8%), Canada (3.9%), and Australia (3.9%). In terms of uploader type, 51.0% of the videos were produced by animation channels, 29.4% by health-related channels, and 19.6% by educational channels. The mean video duration was 198.53 s (median: 179.00), and the mean time since upload was 1630.65 days (median: 1431.00). The mean total number of views was 10,511,577.35 (median: 719,645.00), with a mean of 16,856.94 likes (median: 932.00). The mean viewing rate was 423,946.89 (median: 34,295.54), and the mean interaction index was 0.77 (median: 0.25).

**Table 1:** General characteristics and engagement metrics of the included videos (*n* = 51)

	*n*	%
Language		
Turkish	27	52.9
English	24	47.1
Country of Origin		
Türkiye	27	52.9
Canada	2	3.9
United States	16	31.4
United Kingdom	4	7.8
Australia	2	3.9
Uploader Type		
Animation channel	26	51
Education channel	10	19.6
Health-related channel	15	29.4
	Mean ± SD	Median (Min-Max)
Video duration (seconds)	198.53 ± 125.67	179.00 (60.00–692.00)
Days since upload	1630.65 ± 1137.42	1431.00 (42.00–4873.00)
Total views	10511577.35 ± 37249281.76	719645.00 (174.00–219730678.00)
Number of likes	16856.94 ± 62978.48	932.00 (0.00–426064.00)
Viewing rate	423946.89 ± 1164414.70	34295.54 (7.52–6504756.60)
Interaction index	0.77 ± 1.14	0.25 (0.00–5.14)

Inter-rater reliability results are presented in Table 2. The ICC for the GQS scores was 0.943 (95% CI: 0.902–0.967; *p* < 0.001), and the ICC for the DISCERN scores was 0.906 (95% CI: 0.841–0.945; *p* < 0.001). For the guideline-based content compliance checklist (dichotomous 10-item tool), the ICC was 1.000, indicating complete agreement between the two evaluators.

**Table 2:** Inter-rater reliability of video evaluation scores

	ICC (95% CI)	*p*-value
GQS Score	0.943 (0.902–0.967)	< 0.001
DISCERN Score	0.906 (0.841–0.945)	< 0.001
Guideline-Based Content Compliance Checklist	1.000 (1.000–1.000)	---

*ICC (95% CI)* Intraclass Correlation Coefficient (95% Confidence Interval)

The correlations between evaluation scores are presented in Table 3. The mean GQS score was 2.44 (median: 2.00), the mean DISCERN score was 2.18 (median: 2.00), and the mean guideline-based content compliance score was 4.33 (median: 4.00). Significant positive correlations were observed between the GQS and DISCERN scores (*r* = 0.952; *p* < 0.001), between the GQS and guideline-based compliance scores (*r* = 0.961; *p* < 0.001), and between the DISCERN and guideline-based compliance scores (*r* = 0.926; *p* < 0.001).

**Table 3:** Correlation analysis between evaluation scores

	Mean ± SD	Median (Min-Max)	1	2
1-Mean GQS Score	2.44 ± 1.26	2.00 (1.00–4.00)		
2-Mean DISCERN Score	2.18 ± 1.07	2.00 (1.00–4.00)	0.952 (< 0.001)	
3-Checklist Score	4.33 ± 2.24	4.00 (1.00–8.00)	0.961 (< 0.001)	0.926 (< 0.001)

Values represent Spearman’s rho correlation coefficients (p-values in parentheses)

Comparisons according to video language are presented in Table 4. English-language videos demonstrated significantly higher median GQS scores (3.25 vs. 1.50; *p* = 0.042), DISCERN scores (3.00 vs. 2.00; *p* = 0.037), and guideline-based compliance scores (5.50 vs. 3.00; *p* = 0.018) compared with Turkish-language videos. English-language videos also demonstrated significantly higher total views, number of likes, and viewing rates (all *p* < 0.001). Interaction index values were significantly higher in Turkish-language videos (0.60 vs. 0.18; *p* = 0.003).

**Table 4:** Comparison of quantitative variables according to video language

	Video Language		U	*p*-value	Effect size (*r*)
	Turkish	English			
GQS	1.50 (1.00–4.00)	3.25 (1.00–4.00)	219.000	0.042	0.324
DISCERN	2.00 (1.00–4.00)	3.00 (1.00–4.00)	217.000	0.037	0.330
Checklist Score	3.00 (1.00–7.00)	5.50 (1.00–8.00)	200.500	0.018	0.381
Total views	15961.00 (174.00– 29991674.00)	2238034.50 (37225.00– 219730678.00)	84.000	< 0.001	0.741
Number of likes	58.00 (1.00– 50000.00)	3101.00 (0.00– 426064.00)	112.500	< 0.001	0.653
Viewing rate	3135.69 (7.52– 637171.74)	163265.28 (4283.66–6504756.60)	87.000	< 0.001	0.731
Interaction index	0.60 (0.01–5.14)	0.18 (0.00–1.69)	165.000	0.003	-0.491

Mann-Whitney U test; effect size calculated using rank-biserial correlation (r)

Comparisons of evaluation scores according to uploader type are presented in Table 5. Kruskal–Wallis test results indicated statistically significant differences among animation, educational, and health-related channels regarding the mean GQS, DISCERN, and guideline-based content compliance scores (*p* < 0.001 for all). The effect sizes ranged from moderate to large (η² = 0.254–0.304). Bonferroni-adjusted Dunn post hoc comparisons demonstrated that videos uploaded by animation channels had significantly lower quality and compliance scores compared with those uploaded by both educational and health-related channels. However, no statistically significant differences were observed between educational and health-related channels for any evaluation metrics (Table 5).

**Table 5:** Comparison of GQS, DISCERN, and guideline-based content compliance scores according to uploader type

	Animation Channel	Education Channel	Health-related Channel	Total	Test Statistic	*p*-value	ES [95% CI]
GQS Score	1 (1–4) / 17.92 ^a^	3.5 (1.5–4) / 35.10 ^b^	3.5 (1–4) / 33.93 ^b^	2 (1–4)	16.607	< 0.001^x^	0.304 [0.037–0.692]
DISCERN Score	1 (1–4) / 18.50 ^a^	3 (1–4) / 33.85 ^b^	3 (1–4) / 33.77 ^b^	2 (1–4)	14.435	< 0.001^x^	0.259 [0.014–0.624]
Checklist Score	2.5 (1–7) / 18.46 ^a^	6 (3–8) / 35.05 ^b^	6 (2–8) / 33.03 ^b^	4 (1–8)	14.212	< 0.001^x^	0.254 [0.012–0.617]

^x^ Kruskal–Wallis H test was used. Groups sharing the same superscript letters (a, b) do not differ significantly. Multiple comparisons were performed using the Bonferroni-adjusted Dunn test. Values are presented as median (minimum–maximum) / mean rank. ES [95% CI] = effect size [95% confidence interval]: eta-squared (η²)

No statistically significant correlations were observed between evaluation scores (GQS, DISCERN, and guideline-based compliance) and engagement metrics, including total views, number of likes, viewing rate, and interaction index (*p* > 0.05) (Table 6).

**Table 6:** Correlation between evaluation scores and engagement metrics

	GQS		DISCERN		Checklist Score	
	*r*	*p*-value	*r*	*p*-value	*r*	*p*-value
Total views	-0.027	0.850	-0.002	0.988	-0.029	0.842
Number of likes	0.020	0.891	0.029	0.839	0.007	0.962
Viewing rate	-0.123	0.388	-0.115	0.423	-0.135	0.346
Interaction index	0.128	0.369	0.096	0.501	0.131	0.361

Spearman’s rho correlation coefficients (r) and corresponding p-values

## Discussion

The primary objective of this study was to evaluate the scientific accuracy, educational quality, and guideline adherence of digital toothbrushing education videos intended for children aged 6–12 years. The findings indicate that a substantial proportion of child-oriented oral hygiene content on YouTube does not meet basic educational and evidence-based standards, and that video language is associated with differences in content quality. Of the 210 unique videos screened, only 24.3% (51 videos) fulfilled the inclusion criteria. Most excluded videos consisted of song- or entertainment-based content that did not provide structured, technique-oriented toothbrushing instruction. The relatively small proportion of eligible videos appears to reflect the predominance of entertainment-oriented materials within search results, whereas structured, guideline-based instructional content was less frequently encountered. These findings align with previous investigations evaluating digital health content—including pediatric oral hygiene instructions [13], general dental topics [19, 20], and broader medical fields [21]—which consistently report high exclusion rates and predominantly poor-to-moderate educational quality.

Moreover, the perfect inter-rater agreement observed for the guideline-based content compliance checklist may be explained by the dichotomous structure of the tool. Unlike GQS and DISCERN, which are Likert-type scales requiring subjective grading, the checklist consisted of clearly defined 10 binary (present/absent) items. This structured format may have reduced interpretative variability between evaluators and contributed to complete agreement.

A key finding of the present study is the statistically significant association between video language and quality indicators. Median GQS and DISCERN scores were higher for English-language videos (3.25 and 3.00, respectively) compared with Turkish-language videos (1.50 and 2.00, respectively) (*p* < 0.05). Likewise, the guideline-based compliance score was significantly higher in English-language videos (5.50) than in Turkish-language videos (3.00) (*p* = 0.018). These results suggest that English-language content, on average, may demonstrate closer alignment with evidence-based recommendations. Similar language-related differences have been reported in analyses of gingival recession videos, where English-language content demonstrated higher DISCERN scores than Turkish-language content [22]. This quality disparity may partly reflect differences in platform dynamics and content production ecosystems. English-language videos may benefit from broader audience reach, greater institutional visibility, and more frequent involvement of professional or academic content producers. In contrast, localized-language content may be more dependent on smaller-scale or entertainment-oriented channels, which may contribute to lower guideline adherence. Therefore, the observed language-based differences should be interpreted not only as differences in scientific accuracy but also in relation to broader production and dissemination contexts. Beyond language, the professional background of content creators appears to influence informational quality. In the present study, videos uploaded by animation channels demonstrated significantly lower GQS, DISCERN, and guideline-based compliance scores compared with those originating from educational and health-related channels (*p* < 0.001). This difference suggests that content generated primarily for entertainment or animation purposes may lack scientific accuracy and structured educational value. Consistent with these findings, higher mDISCERN scores have been reported in professionally produced fluoride-related videos compared with amateur content [23]. Studies evaluating oral health education on Instagram have likewise indicated higher usefulness and understandability when materials are prepared by dental professionals [24]. These results align with a growing body of evidence indicating that engagement metrics do not reliably reflect informational accuracy. Previous studies across various dental topics have similarly reported discrepancies between social media popularity and scientific quality, including general oral health misinformation, halitosis, labial frenectomy, and oral hygiene during pregnancy [25–29].

From a clinical perspective, the presence of incomplete or guideline-inconsistent toothbrushing instructions in child-oriented videos is relevant. The 6–12 age period represents a developmental stage in which independent brushing habits and fine motor skills are established. Exposure to inaccurate or incomplete visual models during this period may contribute to the persistence of suboptimal oral hygiene behaviors. Even in videos with moderate overall quality scores, omission of specific instructional elements—such as tongue cleaning—may limit educational effectiveness [15]. Although social media platforms offer opportunities to enhance oral health literacy among children and adolescents, unregulated content may also facilitate the spread of inconsistent practices [30].

In light of these findings, the development of engaging, evidence-based educational materials tailored to children appears essential. Video-based edutainment approaches have been shown to improve clinical indices such as the visible plaque index in school-age populations [31]. Animated oral health education tools have also demonstrated reductions in plaque levels among children with autism spectrum disorder [32]. Furthermore, improvements in the quality of dentist-uploaded YouTube content following the COVID-19 period have been reported [33], suggesting that professional involvement may positively influence digital educational standards.

Several limitations should be considered. Only Turkish- and English-language videos were analyzed, limiting generalizability to other languages. Additionally, comparing English and Turkish content introduces a potential confounding factor due to differences in their respective production ecosystems. English, as a global language, generally benefits from broader audience reach, potentially higher production budgets, and greater institutional backing. These structural differences may influence overall video quality and presentation independently of scientific accuracy; therefore, the language-based differences observed in this study should be interpreted with caution. The cross-sectional design reflects engagement metrics at a single time point, despite the dynamic nature of YouTube content [19]. Additionally, this study did not assess the direct clinical impact of these videos on children’s toothbrushing performance. Furthermore, limiting the screening to the first 60 videos per keyword sorted by relevance introduces a potential selection bias. Given YouTube’s dynamic and algorithm-driven nature, search results are constantly modified based on collective engagement metrics and temporal trends. Consequently, while our sample represents the most prominently featured content at a specific time point, it may not fully capture the entire spectrum of available pediatric toothbrushing videos on the platform.

## Conclusion

Although YouTube represents a potentially valuable platform for pediatric toothbrushing education, the overall scientific quality, reliability, and guideline adherence of widely accessible videos were frequently suboptimal in this sample. Engagement metrics such as views and likes were not associated with informational quality, indicating that popularity should not be interpreted as a marker of scientific accuracy. English-language videos demonstrated higher educational quality and guideline adherence than Turkish-language content. Greater participation of dental professionals in producing accessible, guideline-consistent, and age-appropriate digital educational materials may improve the reliability of online resources available to children and caregivers. To translate these findings into practice, clinicians may guide parents and children toward pre-screened, evidence-based video resources during oral hygiene instruction. Educators may incorporate verified dental edutainment materials into early school-based oral health programs to support standardized baseline knowledge. Policymakers and professional dental associations may also consider developing digital quality labels or accreditation mechanisms for verified pediatric oral health channels, helping families identify reliable online information.

## Data Availability

The dataset generated and/or analyzed during the current study (including video characteristics, evaluation scores, and engagement metrics) is available from the corresponding author upon reasonable request.
